# Bacterial Synthesis and Purification of Normal and Mutant Forms of Human FGFR3 Transmembrane Segment

**Published:** 2011

**Authors:** S.A. Goncharuk, M.V. Goncharuk, M.L. Mayzel, D.M. Lesovoy, V.V. Chupin, E.V. Bocharov, A.S. Arseniev, M.P. Kirpichnikov

**Affiliations:** Shemyakin and Ovchinnikov Institute of Bioorganic Chemistry, Russian Academy of Science; Biology Department, Lomonosov Moscow State University

**Keywords:** membrane protein, FGFR, bacterial expression, purification, detergent solubilization, NMR

## Abstract

The fibroblast growth factor receptor 3 (FGFR3) is a protein belonging to the family of receptor tyrosine kinases. FGFR3 plays an important role in human skeletal development. Mutations in this protein, including Gly380Arg or Ala391Glu substitutions in the transmembrane (TM) region, can cause different disorders in bone development. The determination of the spatial structure of the FGFR3 TM domain in a normal protein and in a protein with single Gly380Arg and Ala391Glu mutations is essential in order to understand the mechanisms that control dimerization and signal transduction by receptor tyrosine kinases. The effective system of expression of eukaryotic genes in bacteria and the purification protocol for the production of milligram amounts of both normal TM fragments of FGFR3 and those with single pathogenic mutations Gly380Arg and Ala391Glu, as well as their^15^N- and [^15^N,^13^C]-isotope-labelled derivatives, were described. Each peptide was produced in*Escherichia coli*BL21(DE3)pLysS cells as a C-terminal extension of thioredoxin A. The purification protocol involved immobilized metal affinity chromatography and cation- and anion-exchange chromatography, as well as the fusion protein cleavage with the light subunit of human enterokinase. The efficiency of the incorporation of target peptides into DPC/SDS and DPC/DPG micelles was confirmed using NMR spectroscopy. The described methodology of production of the native FGFR3 TM domain in norma and with single Gly380Arg and Ala391Glu mutations enables one to study their spatial structure using high-resolution heteronuclear NMR spectroscopy.

## INTRODUCTION

The fibroblast growth factor receptor 3 (FGFR3) belongs to the family of receptor tyrosine kinases (RTKs). This protein consists of an extracellular component with three immunoglobulin-like domains, a hydrophobic transmembrane (TM) domain, and an intracellular component with two tyrosine kinase domains. Specific ligands (fibroblast growth factors) and heparin are bound to the immunoglobulin-like domain of FGFR3, thus stabilizing the dimer complex consisting of two receptor molecules and providing signal transduction inside the cell [1, 2]. FGFR3 plays an important role in the processes of human growth and development (both embryonic/neonatal and that in an adult organism). Mutations in this protein may result in various disorders in the development of connective tissues and skeleton [3–5]. FGFR3 has also been known to participate in tumor formation [5, 6]. In particular, Gly380Arg and Ala391Glu mutations in the TM region of FGFR3 cause lethal dysplasia [7] and the Crouzon syndrome with acanthosis nigricans [8], respectively. The Ala391Glu mutation occurs both upon disorders in skeletal development and upon oncogenesis [6]. The Ala391Glu mutation is considered to stabilize FGFR3 dimerization in the cell membrane, resulting in uncontrollable signal transduction and the emergence of a pathology [9, 10]. However, the detailed mechanism of FGFR3 functioning has not been fully revealed. The approach that has been most frequently used in modern structural biology assumes the division of the membrane protein under study into components and studying the individual water-soluble components of the molecule and its TM regions [11–15]. It is extremely important to obtain a high-resolution structure of the native TM domain of human FGFR3 and that of the domain with Gly380Arg or Ala391Glu mutation to understand the mechanisms that control their dimerization and functioning, because these fragments act as the linking units between the extracellular and intracellular RTK domains and directly participate in signal transduction inside the cell.

**Fig. 1:**
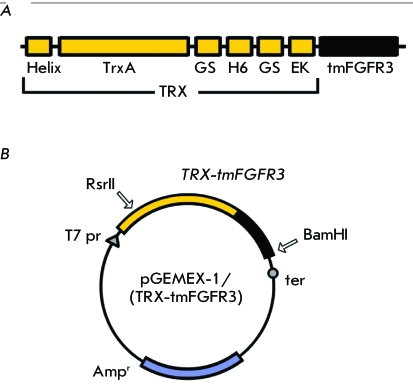
Schematic representation of (A) TRX-tmFGFR3 fusion proteins and (B) expression vectors. Helix – N-terminal fragment of a membrane-active protein from *Helicobacter pylori* ; TrxA – thioredoxin A of *E.coli* ; GS – GlySerGlySerGly aminoacid sequence; H6 – hexahistidine sequence; EK – enterokinase light chain cleavage site; tmFGFR3 – target transmembrane peptide from FGFR3 in norma or with Gly380Arg and Ala391Glu single point mutations; *Amp ^r^* – ampicillin resistance gene.

In this paper, an efficient system of gene expression and purification protocol are described which enable one to produce preparative amounts of the FGFR3 TM fragment both in norma and with single Gly380Arg and Ala391Glu mutations, as well as their ^15^ N- and [ ^15^ N, ^13^ C]-labelled derivatives. The designed approach of producing TM peptides facilitates the study of their structure by high-resolution heteronuclear NMR spectroscopy.

## EXPERIMENTAL

In this study, we used *Escherichia coli* strains XL-10-Gold (Stratagene, United States) and BL21(DE3)pLysS (Stratagene, United States), plasmids pGEMEX-1 (Promega, United States) and pGEMEX-1/TRX-TMS [16]. Oligonucleotides were synthesized by Evrogen (Russia). DNA was sequenced at the Inter-institute Center of Shared Use GENOME (Russia). The reagents purchased from CIL (United States) were used to introduce the isotope labels ^15^ N and ^13^ C. The completely deuterized dodecylphosphoglycerol (DPG) was produced by enzymatic transphosphatidylation from completely deuterized dodecylphosphocholine (DPC) and glycerol in the presence of phospholipase D [17].

**Gene cloning**

Plasmid vectors for the expression of peptide genes as fusion proteins with thioredoxin A were constructed as described previously [12, 16–19]. The genes encoding TM fragments of human FGFR3 ( *tmFGFR3* ) (amino acid residues 357–399 of the normal FGFR3 ( *tmFGFR3-nat* ) and 357–399 of FGFR3 with point mutations G380R ( *tmFGFR3-R* ), and A391E ( *tmFGFR3-E* )) were assembled using six chemically synthesized oligonucleotides with partially overlapping nucleotide sequences. The codons used were optimized for the gene expression in *E. coli * cells. The restriction site BamHI and the sequence encoding the enterokinase recognition site were introduced into the 3’ and 5’ terminal primers, respectively. The same sequence was added to the 3’ terminus of the carrier protein gene ( *TRX* ) amplified using PCR from the pGEMEX-1/(TRX-TMS) vector [16]. The recombination of the *TRX* and *tmFGFR3 * genes was performed using PCR yielding *TRX-tmFGFR3. * The expression plasmids pGEMEX-1/(TRX-tmFGFR3) ( *Fig. 1B* ) were obtained by cloning *TRX-tmFGFR3* fragments treated with RsrII and BamHI restriction endonucleases into pGEMEX-1/(TRX-TMS) vectors linearized with the same proteases [16]. The validity of the nucleotide sequence within expression cassettes was confirmed by DNA sequencing on both strands.

**Selection of cultivation conditions for the recombinant**

*E. coli* **strain**

Fusion protein genes were expressed in the *E. coli* cells BL21(DE3)pLysS. The cells were cultured in rich and minimal media; both chemical induction of protein synthesis (TB and M9 media) and autoinduction [20] (media BYM5052, М5052, С750501’, or M50501, *Table 1* ) being used. When selecting the optimal conditions for protein synthesis, an inducing agent, isopropyl-β-D-thiogalactoside (IPTG), was added into the cell culture that was cultivated at 28°С and attained the optical density of *ОD*_550 _ ~1.5 AU (TB medium) or ~0.6 AU (M9 medium) up to the final concentrations of 1, 0.25, 0.05, 0.01, and 0 mM. Cultivation was continued for 15 h at 250 rpm and a temperature of 37°С; for 40 h (TB) or 60 h (M9) at 25°С; and 60 h (TB) or 72 h (M9) at 13°С. In the case of autoinduction media, the cells were cultivated at 300 rpm and a temperature of 18°С for 4 (BYM5052) or 7 days (М5052, С750501’, or M50501). The optimal temperature, IPTG concentration, and cultivation time were determined using Tris-glycine SDS-PAGE electrophoresis.

**Gene expression**

**Table 1:** Composition of the auto-induction media used

Medium	Studier^a^	Na_2_HPO_4_(mM)	KH_2_PO_4_(mM)	Bacto trypton	Yeast extract, %	Glycerol	Na_2_SO_4_, MgSO_4_, NH_4_Cl, Glucose, Lactose, Metals
BYM5052	ZYM-5052	+	+	2%	1%	+	+
М5052	N-5052	25	25		0.0002%	+	+
C750501’	C-750501	+	+		0.0002%	+	+
M50501	C-750501	25	25		0.0002%	0.5%	+

Auto-induction medium that is taken as a basis.

Note: Components with a concentration equal to the one used by Studier [20] are marked with a + sign.

A M9 medium containing 0.0002% of yeast extract, ^15^ NH _4_ Cl, and [U- ^13^ С]-glucose ( ^15^ N, ^13^ C-labelling) or  ^15^ NH _4_ Cl and nonenriched glucose ( ^15^ N-labeling) was used for the preparative obtaining of labelled proteins. In order to produce target fusion proteins, IPTG was added into the cell culture with * OD*_600_ ~ 0.6 AU (M9 medium, isotope labelling) or 1.5 AU (TB medium, no labelling) up to a final concentration of 0.05 mM and the temperature was reduced from 28 to 13°С. The cells were cultivated at 250 rpm for 72 h. The cells were then harvested and stored at –20°C.

**Target protein purification**

The biomass obtained from 1 L of the culture was suspended in 50 ml of lysing buffer (50 mM Tris, pH 8.0, 150 mM NaCl, 10 mM imidazole, 1% Triton X-100, 0.2 mM phenylmethylsulfonyl fluoride), destroyed by ultrasound, centrifuged, and filtered through a membrane (pore size 0.22 µm). The clarified lysate was applied to a column with Chelating Sepharose FF (Amersham Bioscience, United States) preliminarily charged with Ni ^2+ ^ and balanced with buffer A (50 mM Tris, pH 8.0, 250 mM NaCl, 1% Triton Х-100) containing 10 mM imidazole. The resin was successively washed with buffer A containing 10 mM imidazole and the same buffer containing 40 mM imidazole. The protein was eluted with buffer A containing 175 mM imidazole. The eluate was diluted by a factor of 11 with buffer containing 17 mM Tris, pH 8.0, 20 mM NaCl, and 1% Triton Х-100; then, the light chain of recombinant human enterokinase was added [21] at a ratio of 25 units of enzyme per 1 mg of TRX-tmFGFR3. The mixture was incubated for a night at room temperature and applied to a column with Chelating Sepharose FF balanced with buffer B, pH 8.0 (20 mM Tris, 40 mM NaCl, 1% Triton Х-100, 16 mM imidazole). The unbound to the resin fraction was collected, pH was decreased to 4.55 using concentrated acetic acid, filtered through a membrane (pore size 0.22 µm), and applied to a column with SP Sepharose FF (Amersham Bioscience, United States) balanced with buffer B, pH 4.55. After the fraction was applied, the resin was washed with the same buffer. The unbound to the resin fraction was collected, the pH was brought to 9.0 using NaOH, filtered through the membrane (pore size 0.22 µm), and applied to a column with Q Sepharose FF (Amersham Bioscience, United States) balanced with buffer C (20 mM Tris, рН 8.8, 1% Triton Х-100). The peptides were eluted with a linear NaCl gradient (0–1 M). After incubation with a 10% trichloroacetic acid (TCA) solution, the purified peptides were washed thrice with acetone and vacuum-dried. The purity and identity of the purified peptides to the target ones were confirmed by gel electrophoresis, MALDI mass spectroscopy (Daltonics Ultraflex II TOF/TOF, Bruker Daltonik, Germany), and NMR spectroscopy.

**Solubilization of tmFGFR3 in a membrane-like environment**

For preliminary folding into a helical conformation the specimens of isotope labelled tmFGFR3 were dissolved in a TFE/H _2_ O (60/40) mixture with 2 mM tris(2-carboxyethyl)phosphine (TCEP) added in order to prevent the formation of nonspecific intermolecular disulfide bonds. Complete solubilization was achieved using 10 freeze (in liquid nitrogen)/thaw cycles. Homogenized specimens were obtained under ultrasonication (ultrasonic bath D-78224 Singen/Htw (Elma, Germany)) at the thaw stage in each cycle. The solubility and formation of the secondary structure of tmFGFR3 in the TFE/H _2_ O mixture was controlled using ^1^ H/ ^15^ N-bestHSQC NMR spectra [22–24] by analyzing the signal width and signal dispersion. A solution of tmFGFR3 in TFE/H _2_ O was mixed with the necessary amount of detergents and/or lipids dissolved in TFE/H _2_ O to obtain a detergent (lipid)/peptide ratio ranging from 120 to 40. The resulting mixture was lyophilized (ModulyoD-230 Freeze Dryer, Thermo, Canada) and dissolved in H _2_ O/D _2_ O (10/1) with 10 freeze/thaw cycles (under ultrasonic action) to attain protein homogeneity and complete incorporation into detergent micelles or lipid bicelles. Heteronuclear NMR spectra of tmFGFR3 peptides incorporated into supramolecular complexes were obtained at 40°С, the pH varied from 3.5 to 6.5 on an AVANCE spectrometer (Bruker, Germany) equipped with a cryogenically cooled high-sensitivity sensor, with a proton operating frequency of 700 MHz.

## RESULTS AND DISCUSSION

**System of**

**Table 2:** Efficiency of the method of fusion proteins (TRX-tmFGFR3) and target peptides (tmFGFR3) production

TM-peptide	Molecular weight, kDa	Aminoacid sequence of a TM fragment^a^	EK^b^, u/mg	Yield^c^, mg/ml	
				TRX-tmFGFR3	tmFGFR3
tmFGFR3-nat	4.6	L^357^PAEEELVEADEAGSVYAGILSYGVGFFLFILVVAAVTLCRLR^399^	25	40	6
tmFGFR3-R	4.7	L^357^PAEEELVEADEAGSVYAGILSYR^380^VGFFLFILVVAAVTLCRLR^399^	30	20	4
tmFGFR3-E	4.7	L^357^PAEEELVEADEAGSVYAGILSYGVGFFLFILVVE^391^AVTLCRLR^399^	30	50	7

The putative TM domains are indicated as gray boxes. The point mutations Gly380Arg (tmFGFR3-R) and Ala391Glu (tmFGFR3-E) appear in bold.

Activity of the enterokinase light chain required to hydrolyze 1 mg of TRX-tmFGFR3 fusion proteins.

The average yield (per 1 L of bacterial culture in M9 minimal media) of the fusion proteins (TRX-tmFGFR3) and purified peptides (tmFGFR3), including their ^15^ N- and [ ^15^ N-, ^13^ C]-labelled derivatives. The yields were estimated by the intensity of the Coomassie blue-stained bands in SDS-PAGE and by weighing pure, dried tmFGFR3 peptides.

*tmFGFR3* **gene expression**

Peptides with a primary structure corresponding to the full-length TM fragment of FGFR3 (tmFGFR3) with regions adjacent to the hydrophobic fragment (normal, tmFGFR3-nat and that with pathogenic point mutations G380R (tmFGFR3-R) or A391E (tmFGFR3-E)) were studied in this work ( *Table 2* ). 

Due to the rapid proteolytic degradation of small peptides during their expression in bacterial cells, tmFGFR3 was obtained as thioredoxin A (TrxA) fusion protein, as described earlier [12] ( *Fig. 1A* ). Six histidine residues (H6), the recognition site of the human enterokinase light chain (EK), and mobile glycine-rich fragments Gly-Ser-Gly-Ser-Gly (GS) on both sides from H6 were incorporated between the TrxA and tmFGFR3 fragments of the fusion protein. The highly specific enzyme EK selectively hydrolyzes the peptide bond located immediately after the recognition site (with the exception of the Lys-Pro bond). The amino acid sequence Helix is located at the N terminus of the fusion protein [12], facilitating the elimination of the toxicity of some TM peptides with respect to the host cell (no data are provided). The genes encoding the fusion proteins Helix-TrxA-GS-H6-GS-EK-tmFGFR3 (hereinafter referred to as TRX-tmFGFR3) were incorporated into pGEMEX-1 plasmid vectors under the transcriptional control of the Т7 promoter yielding pGEMEX-1/(TRX-tmFGFR3) expression vectors ( *Fig. 1B* ).

To produce proteins, *E. coli* BL21(DE3)pLysS cells were used, since an acceptable level of expression of target genes can be provided by these cells. When selecting between autoinduction [20] and chemical induction, the choice in favor of the former is justified by the absence of the necessity to add an inducing agent when the cell culture attains a certain optical density. The yields of the target proteins being comparable, the induction by IPTG wins out economically, since [U- ^13^ C]-glucose can be used as the only carbon source, instead of the more expensive [U- ^13^ C]-glycerol for ^13^ C isotope labelling. 

**Fig. 2:**
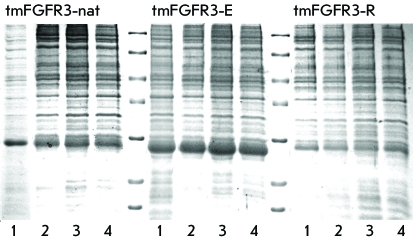
Efficiency of production of TRX-tmFGFR3 fusion proteins in M9 minimal salt and М5052, С750501’ and M50501 auto-induction media. Coomassie blue-stained 14% Tris-glycine SDS-PAGE shows the fractionation of the lysate of whole cells producing TRX-tmFGFR3-nat, TRX-tmFGFR3-E and TRX-tmFGFR3-R. Recombinant strains were grown in: *1* – M9 medium, 13°C after induction with 0.05 mM IPTG; *2 –* M5052 auto-induction medium, 18°C; *3 –* M50501 auto-induction medium, 18°C; and *4* – С750501’ auto-induction medium, 18°C. Equivalents of 20 μL of cell culture were loaded into each lane. Protein molecular weight markers: 116.0, 66.2, 45.0, 35.0, 25.0, 18.4, and 14.4 kDa (top-down).

The cultivation conditions at which maximum accumulation of the target proteins was observed were determined by testing the media used for protein synthesis induction using IPTG (TB and M9), as well as the auto-induction media proposed by FW Studier [20], taken with certain modifications ( *Table 1* ). The yeast extract was added to the media intended for the production of isotope-labelled peptide derivatives (M5052 – to introduce ^15^ N, and M9, С750501’, and M50501 – to introduce [ ^15^ N, ^13^ С]) to obtain a concentration of 0.0002%. It was shown experimentally that this concentration of the yeast extract promotes the maximum increase in the yield of the target protein without having an effect on the ^15^ N and  ^13^ С incorporation in the target protein. Considering the high cost of [U- ^13^ C]-glycerol, we carried out a number of experiments to determine the optimal glycerol concentration in the auto-induction medium at which maximum accumulation of the target product was observed. It appeared that a decrease in glycerol concentration by a factor of 1.5, along with a twofold fall in phosphate concentration in the culture medium (M50501 medium), either has no effect on the yield of the target products (tmFGFR3-nat and tmFGFR3-R) or enhances its accumulation (tmFGFR3-E) ( *Fig. 2* ). This fact makes it possible to considerably reduce the cost of the production of [ ^15^ N, ^13^ С]-labelled preparations by using the auto-induction principle.

**Fig. 3:**
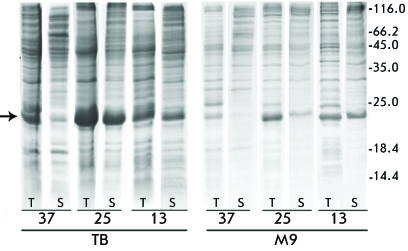
Efficiency of production of TRX-tmFGFR3-E target fusion proteins in rich (TB) and minimal (M9) media depending on growth temperature (37°C, 25°C, 13°C) after IPTG induction. Coomassie blue-stained 14% Tris-glycine SDS-PAGE analysis of TRX-tmFGFR3-E cell lysate (0.05 mM IPTG). Protein molecular weight markers (kDa) are shown on the right. The arrow on the left indicates TRX-tmFGFR3-E target fusion protein. 5 (TB) or 10 μL (M9) of cell culture were loaded into each lane. T — total cellular protein; S — soluble protein fraction.

Bacterial cells transformed with the appropriate vector were cultivated at 18°С in the case of auto-induction; or at 37, 25, and 13°С (after IPTG was added) when using chemical induction. The decrease in the cultivation temperature promotes maintenance of the protein in soluble form [12]. Thus, in the case of chemical induction, when cultivating cells both in rich (TB) and minimal (M9) media at high temperature (37°С), after adding IPTG, the fusion proteins mostly accumulated within inclusion bodies. With the temperature decreasing to 25°С, protein solubility increased; the inclusion bodies contained half of the protein. At 13°С, all fusion proteins were observed mostly in soluble form ( *Figs. 2 and 3* ).

The dependence of the gene expression level on the cultivation temperature or concentration of the inducing agent in case of chemical induction (1.0, 0.25, 0.05, and 0.01 mM IPTG) was assessed using SDS-PAGE electrophoresis. Based on the analysis results, a rich TB medium was used to produce target proteins (13°С after the induction) without incorporation of isotope labels. When using the M9 and M50501 media, the yield of fusion proteins appeared to be comparable ( *Fig. 2* ); therefore, the M9 medium (13°С after the induction) was selected for the production of preparative amounts of isotope-labelled target proteins. A maximum yield of all TRX-tmFGFR3 or their ^15^ N- or [ ^15^ N, ^13^ C]-labelled derivatives was attained at 0.05 mM IPTG.

**Fusion protein purification**

**Fig. 4:**
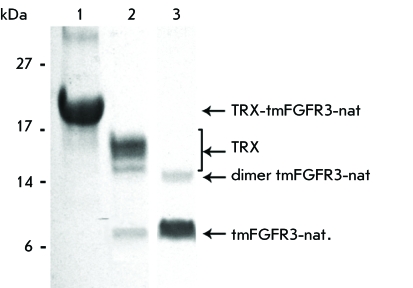
Efficiency of purification of tmFGFR3-nat: *1* – purified fusion protein, *2* – products of enterokinase cleavage, *3* – purified tmFGFR3-nat. Arrows on the right indicate: TRX-tmFGFR3-nat fusion protein, TRX fusion partner, tmFGFR3-nat dimer and monomer compounds. Coomassie blue-stained 14% Tricine SDS-PAGE. Calculated molecular weights: TRX-tmFGFR3-nat – 19.6 kDa, tmFGFR3-nat – 4.6 kDa.

After cell lysis, fusion proteins were purified using immobilized metal affinity chromatography (IMAC). In order to prevent the precipitation of target proteins, the non-ionic detergent Triton X-100 was used at this or subsequent purification stages. The purity of the protein preparations obtained by IMAC was at least 80%. The molecular weights of the fusion proteins determined on the basis of their electrophoretic mobility (SDS–PAGE, tricine buffer) ( *Fig. 4* ) were similar to the calculated values.

The fusion proteins TRX-tmFGFR3 purified by IMAC were cleaved using the human enterokinase light chain (EK) [21] ( *Fig. 1A* ). When optimizing the reaction conditions for each peptide, the efficiency of the subsequent purification studies was accounted for. An optimal composition of the reaction mixture was obtained by diluting the fractions containing the fusion protein by a factor of 11 (see the EXPERIMENTAL section). EK (30 units per 1 mg of fusion protein TRX-tmFGFR3) was used for complete isolation of tmFGFR3 peptides from the partner protein ( *Fig. 4* ).

**Fig. 5:**
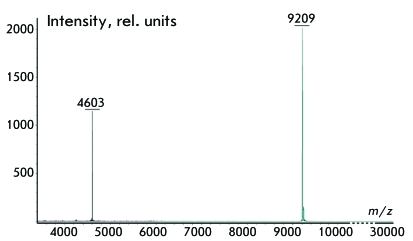
Results of mass-spectroscopy analysis of the purified tmFGFR3-nat. The peaks in the spectrum correspond to tmFGFR3-nat monomer (m/z 4603) and dimer (m/z 9209) compounds.

After the fusion protein had been cleaved for a night, IMAC was performed to remove the TRX fragment and the residual amounts of fusion proteins from the reaction mixture. The concentration and additional purification of tmFGFR3-target peptides using two successive stages of cation-exchange and anion-exchange chromatography at pH values ensuring the maximum charge and affinity of the target polypeptides towards ion-exchange resins were used to obtain protein preparations with a purity of at least 97%. The results of SDS–PAGE electrophoresis attest to the efficiency of TRX-tmFGFR3-E hydrolysis and tmFGFR3-E purification ( *Fig. 4* ). The data on the purification and efficiency of the proposed protocol for tmFGFR3-nat and tmFGFR3-R are identical. The electrophoretical mobility of tmFGFR3 corresponds to that of peptides mostly in monomeric conformations. The purity and correspondence of the purified peptides to the target tmFGFR3 were confirmed by mass spectroscopy analysis ( *Fig. 5* ) and NMR spectroscopy.

As mentioned above, tmFGFR3 peptides were obtained in the presence of Triton X-100. The high optical density of the aqueous solution of Triton X-100 impedes the use of the optical methods of analysis and determination of the secondary peptide’s structure in this detergent using CD spectroscopy. In the case of NMR spectroscopy (see below), even trace amounts of Triton X-100 in the sample had a negative effect on the properties of the membrane-like environment that was used for structural studies, as well as the spatial structure of the protein. Peptides with Triton X-100 were precipitated with TCA, followed by washing of the precipitate with cooled acetone, in order to efficiently remove the detergent from the solution [12]. High efficiency of Triton X-100 removal from protein samples was confirmed by NMR spectroscopy. Using the procedure described, the yield of target proteins was brought up to 4–8 mg/l of the culture. The purity of the recombinant proteins and the degree of [ ^15^ N, ^13^ С]-label incorporation were at least 97%.

**Solubilization of tmFGFR3 in the membrane-like environment**

The selection of a medium imitating the surroundings of an object in the cell membrane is of exceptional significance for a successful study of structure and functions [23]. The composition of a membrane-like environment that would be optimal for NMR studies is determined by the following main parameters: size of supramolecular particles with tmFGFR3 incorporated into them; sample monodispersity; the absence of aggregation and sample stability; implementation of the native helical conformation; and tmFGFR3 dimerization. How closely the tmFGFR3 supramolecular complexes in the selected membrane-like environment met these criteria was estimated using NMR spectroscopy. Both detergent micelles and lipid bicelles of different compositions were used as membrane-like media. The total quality of the samples in terms of the possibility of carrying out further structural studies by NMR spectroscopy was assessed using two-dimensional spectra ^1^ H/ ^15^ N-bestHSQC and  ^1^ H/ ^15^ N-TROSY. The total number of allowed cross-peaks within the region of the NH-signals of glycerol residues, dispersion, broadening, and signal doubling were analyzed.

It should be noted that both zwitterionic and charged deuterized detergents, which provide a possibility of imitating partially charged cell membranes, are often required to perform structural studies of membrane proteins and peptides by NMR methods. Today, SDS is the only commercially available detergent that is completely deuterized and negatively charged. This detergent has no structural analogues among the phospholipids that are components of biological membranes; therefore, the use of SDS to simulate membrane properties is not always reasonable. In this study, we made an attempt to use completely deuterized DPG synthesized by us, in order to generate a partially negative charge on the micellar surface. The structure of the polar head of DPG is identical to that of phosphatidylglycerol, the main negatively charged phospholipid within bacterial membranes. The use of DPG allows to better simulate the properties of biological membranes as compared with SDS.

**Fig. 6:**
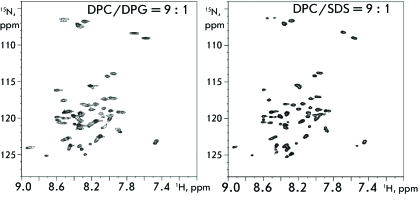
^1^ H/ ^15^ N-bestHSQC NMR spectra of tmFGFR3-nat in DPC/DPG (left) and DPC/SDS (right) micelles. Temperature is 40°С, pH 5.7, and detergent/peptide molar ratio is 40.

We selected tmFGFR3-nat solubilization conditions, which made it possible to study its spatial structure and dimerization. The best results upon solubilization of the tmFGFR3-nat peptide were obtained when using the mixed micelles of completely deuterized DPC/DPG (9/1 mol/mol) and DPC/SDS (9/1 mol/mol). The total number of peaks, good signal dispersion (being considerably higher than the corresponding values for the peptide in random conformation, which attests to the formation of the secondary structure), and small line width in the ^1^ H/ ^15^ N-bestHSQC spectrum totally correspond to its secondary structure and the hydrodynamic size expected based on the amino acid sequence of the peptide ( *Fig. 6* ). The presence of cross-peak doubling in ^1^ H/ ^15^ N-bestHSQC spectra, as well as the dependence of the relative intensities in these doublets on the number of tmFGFR3-nat molecules incorporated in one micelle ( *Fig. 6* ), points to the successful determination of the tmFGFR3-nat dimerization conditions that are suitable for structural studies using heteronuclear NMR spectroscopy.

## CONCLUSIONS

The elaborated system of gene expression and purification protocol enables to produce recombinant transmembrane peptides tmFGFR3, including the isotope labelled derivatives to milligram amount, which are required for structural and functional studies. The relatively small size of the peptide complexes in the membrane-like environment attests to the possibility of obtaining the spatial structure of tmFGFR3-nat in dimeric state using high-resolution heteronuclear NMR spectroscopy [12, 24]. The conformation of the tmFGFR3-nat dimer was determined recently, and the study of the processes accompanying the specific association of tmFGFR3-E and tmFGFR3-R is now under way. The proposed technology of recombinant peptides production will help better understand the mechanism underlying the functioning, as well as signal transduction, with the participation of the FGFR3 receptor, as well as shed light on the molecular mechanisms of different disorders in human skeletal development, wich are directly associated with mutations in the FGFR3 TM domain.

## Abbreviations

FGFR - Fibroblast growth factor receptor family; FGFR3 - Fibroblast growth factor receptor 3; RTK - receptor tyrosine kinase; TM - transmembrane (domain of membrane protein); DPC: dodecylphosphocholine
DPG: dodecylphosphoglycerol
SDS - sodium dodecyl sulfate; TFE: 2,2,2-trifluoroethanol
